# A Transcriptomic Approach Provides Insights on the Mycorrhizal Symbiosis of the Mediterranean Orchid *Limodorum abortivum* in Nature

**DOI:** 10.3390/plants10020251

**Published:** 2021-01-28

**Authors:** Rafael B. S. Valadares, Fabio Marroni, Fabiano Sillo, Renato R. M. Oliveira, Raffaella Balestrini, Silvia Perotto

**Affiliations:** 1 Instituto Tecnológico Vale, Rua Boaventura da Silva 955, 66050-000 Belém, Pará, Brazil; rafaelbsvaladares@gmail.com (R.B.S.V.); renato.renison@gmail.com (R.R.M.O.); 2 Dipartimento di Scienze Agroalimentari, Ambientali e Animali, Università di Udine, Via delle Scienze, I-33100 Udine, Italy; marroni@appliedgenomics.org; 3 Istituto di Genomica Applicata, Via Linussio 51, I-33100 Udine, Italy; 4 Consiglio Nazionale Delle Ricerche-Istituto per la Protezione Sostenibile Delle Piante, Viale P.A. Mattioli 25, I-10125 Torino, Italy; fabiano.sillo@ipsp.cnr.it; 5 Instituto de Ciências Biológicas, Universidade Federal de Minas Gerais, Av. Pres. Antônio Carlos, 6627, 31270-901 Belo Horizonte, Minas Gerais, Brazil; 6 Dipartimento di Scienze della Vita e Biologia dei Sistemi, Università di Torino, Viale Mattioli 25, I-10125 Torino, Italy

**Keywords:** orchid mycorrhiza, transcriptomics, nitrogen metabolism, CAZymes

## Abstract

The study of orchid mycorrhizal interactions is particularly complex because of the peculiar life cycle of these plants and their diverse trophic strategies. Here, transcriptomics has been applied to investigate gene expression in the mycorrhizal roots of *Limodorum abortivum*, a terrestrial mixotrophic orchid that associates with ectomycorrhizal fungi in the genus *Russula*. Our results provide new insights into the mechanisms underlying plant–fungus interactions in adult orchids in nature and in particular into the plant responses to the mycorrhizal symbiont(s) in the roots of mixotrophic orchids. Our results indicate that amino acids may represent the main nitrogen source in mycorrhizal roots of *L. abortivum*, as already suggested for orchid protocorms and other orchid species. The upregulation, in mycorrhizal *L. abortivum* roots, of some symbiotic molecular marker genes identified in mycorrhizal roots from other orchids as well as in arbuscular mycorrhiza, may mirror a common core of plant genes involved in endomycorrhizal symbioses. Further efforts will be required to understand whether the specificities of orchid mycorrhiza depend on fine-tuned regulation of these common components, or whether specific additional genes are involved.

## 1. Introduction

Mycorrhizas are among the most widespread plant–fungus symbioses on Earth. Different mycorrhizal types have been described in terrestrial plants, depending on the taxonomic nature of the partners and their interactions [1]. In ectomycorrhiza, the fungal hyphae remain outside the plant cell wall boundaries, whereas in endomycorrhiza—which includes arbuscular, ericoid, and orchid mycorrhiza—the symbiosis culminates in the formation of intracellular fungal structures. Endomycorrhiza formation is a complex multistep process where the exchange of molecular signals between mycorrhizal fungi and their host plants during the extra-radical phase is followed by close surface-to-surface interactions and accommodation of the fungus inside the root cells [2]. Due to their wide distribution and occurrence in economically important crops, the molecular bases of mycorrhizal fungus–host plant interactions have been investigated mainly in arbuscular mycorrhiza (AM). Plant metabolism is deeply influenced by the AM symbiosis, with many upregulated genes being related with nutrient transport, cell wall metabolism, and potential involvement in the accommodation of the AM fungus, as well as genes encoding for transcription factors [3,4]. As compared to the AM symbiosis, little is known on the metabolic changes occurring in orchid mycorrhiza (OM), despite the fact that orchids belong to one of the largest families of flowering plants, counting about 28,000 species distributed worldwide and including terrestrial species, epiphytes, and geophytes [5]. 

The study of OM interactions is particularly complex because of the peculiar life cycle of orchids and their diverse trophic strategies. In fact, germination of the minute endosperm-lacking seeds leads to the formation of a tuber-like structure named protocorm [6,7]. Protocorms are fully heterotrophic in most orchid species and are provided with carbon and other nutrients by the mycorrhizal fungal partners, a strategy named mycoheterotrophy [8]. Protocorms eventually give rise to leaflets and true seedlings that may grow into adult plants with green photosynthetic leaves. However, adult plants remain achlorophyllous in about 200 orchid species, and keep relying fully on fungal derived carbon [8]. Many forest orchids develop green leaves but nevertheless remain partially dependent on the fungal partner for carbon, a strategy termed mixotrophy [9]. Irrespective of the trophic strategies, all stages of the orchid life cycle are mycorrhizal in nature, and orchids are thought to be strictly dependent on their symbiotic fungal partners for growth and survival, starting from the germinating seeds through to adulthood [10]. With few exceptions, the taxonomic position and ecological role of OM fungi closely mirrors the trophic strategy of the adult host plant. Most photosynthetic orchids associate with fungi in the “rhizoctonia” complex, an assemblage of species taxonomically assigned to Agaricomycetes in the orders Cantharellales (Tulasnellaceae and Ceratobasidiaceae) and Sebacinales (Serendipitaceae). By contrast, most mycoheterotrophic and mixotrophic orchids associate with Basidiomycetes and Ascomycetes known to form ectomycorrhiza (ECM) on other host plants [11]. The association with ECM fungi allows these parasitic orchids to ultimately gain carbon from photoautotrophic plants through the common mycorrhizal network that connects the roots of ECM plants in forest communities [12,13]. 

Metabolic changes in OM tissues have been investigated mainly in the protocorms of photosynthetic orchid species, colonized in vitro by compatible rhizoctonia-like fungi. Early studies described in Smith and Read [1] already revealed transfer of organic carbon from the fungal partner to *Dactylorhiza purpurella* protocorms, and were confirmed by more recent studies in *Spiranthes sinensis* [14]. Transcriptomic [15,16,17] and proteomic [18,19,20] approaches have revealed the upregulation of several plant membrane transporters in mycorrhizal protocorms, including amino acid transporters and bidirectional sugar transporters of the SWEET (Sugars Will Eventually be Exported Transporters) family [15,21,22]. Interestingly, a recent metabolomic study revealed that the external mycelium of the mycorrhizal fungus *Tulasnella calospora* growing near the host protocorms showed significant metabolic changes, mainly in lipid metabolism [23].

Thus, orchid protocorms have been instrumental to reveal molecular changes in OM interactions. However, these tuber-like structures are anatomically and metabolically very different from the roots of adult orchids, especially in photosynthetic or mixotrophic species. We actually know very little on the metabolic and molecular changes occurring in the mycorrhizal roots of adult orchids. Although Cameron et al. [24,25] demonstrated a transfer of N and P from the fungal partner to the host plant *Goodyera repens*, actual mutualism is still debated even for photosynthetic orchid species, and a net flow of plant-derived fixed C to the mycorrhizal fungus has been so far demonstrated in vitro only for two photosynthetic orchid species, *G. repens* [24,26] and *Serapias strictiflora* [27].

Molecular studies on mycorrhizal roots of adult orchids are very few. A transcriptomic approach has been used to investigate changes in gene expression in adult OM roots of the epiphytic photosynthetic orchid *Cymbidium hybridum*, inoculated in vitro with the mycorrhizal fungus *Epulorhiza repens* [16]. To our knowledge, *Oeceoclades maculata* is the only orchid species where gene and protein expression in mycorrhizal and non-mycorrhizal roots was compared under natural conditions [28]. This tropical terrestrial orchid specifically associates with the wood decomposer *Psathyrella candolleana* [28], a fungus found as symbiont of mycoheterotrophic orchids [29]. Suetsugu et al. [30] also used RNAseq to investigate gene expression in mycorrhizal roots of adult orchids in nature, but their comparison of albino and green variants of *Epipactis helleborine* aimed to uncover the molecular mechanisms related to mycoheterotrophy, rather than to OM interactions. 

Here, we used a transcriptomic approach to investigate changes in gene expression in the mycorrhizal roots of adult individuals of *Limodorum abortivum* grown in nature, as compared to non-mycorrhizal roots. We focused on *L. abortivum* because this terrestrial orchid form very thick roots with a large cortical parenchyma usually colonized by intracellular fungal coils, but non-mycorrhizal roots can be found in nature and were used as reference. In addition, previous studies on *L. abortivum* provide useful information on plant metabolism and fungal diversity. Stable isotope profiling [31] as well as biochemical and physiological analyses have in fact demonstrated that *L. abortivum* is a mixotrophic orchid with very inefficient photosynthesis and a specific association with ECM fungi in the genus *Russula* [32]. 

This study on *L. abortivum* expands our knowledge on the molecular bases of mycorrhizal interactions in adult orchids in nature, and addresses for the first time OM interactions in a species associated with an ECM fungus. Additionally, the comparison with a nonsterile non-mycorrhizal condition as reference likely facilitated the identification of mycorrhiza-specific plant responses over general plant responses to soil and root-associated microorganisms.

## 2. Results

### 2.1. Analysis of RNA-Seq Data

The number of raw and trimmed reads, together with the number of uniquely aligned reads, are shown in Table S1. Only reads uniquely aligned on the assembled transcriptome were used for differential expression analyses, corresponding to 4,220,880 reads (SD = 287,707) for mycorrhizal roots (LM), and 4,518,992 reads (SD = 501,399) for non-mycorrhizal roots (LS) samples. Reads assigned to fungi were on average 695,978 (SD = 400,597) in LM, and 190,961 (SD = 58,173) in LS. 

A total of 30,591 transcripts tested for differential expression (DE) by DESeq2 [33] were assigned to Streptophyta. The 10 plant taxa identified by Taxon kit and represented by the highest number of transcripts are reported in Figure S1. The genome of *L. abortivum* has not been sequenced, but about 65% of plant transcripts matched with other orchid genera, namely *Dendrobium*, *Phalaenopsis*, *Corallorhiza*, and *Neottia*. Of the genes assigned to Streptophyta, 1677 were differentially expressed in LM and LS. In detail, when compared to LS samples, 1167 DE genes out of 1677 were upregulated in LM, while 510 were found to be downregulated (Table S2). The number of fungal transcripts identified (3094) was sensibly lower. 

### 2.2. Transcriptomic Profile of L. abortivum Roots

**Figure 1:**
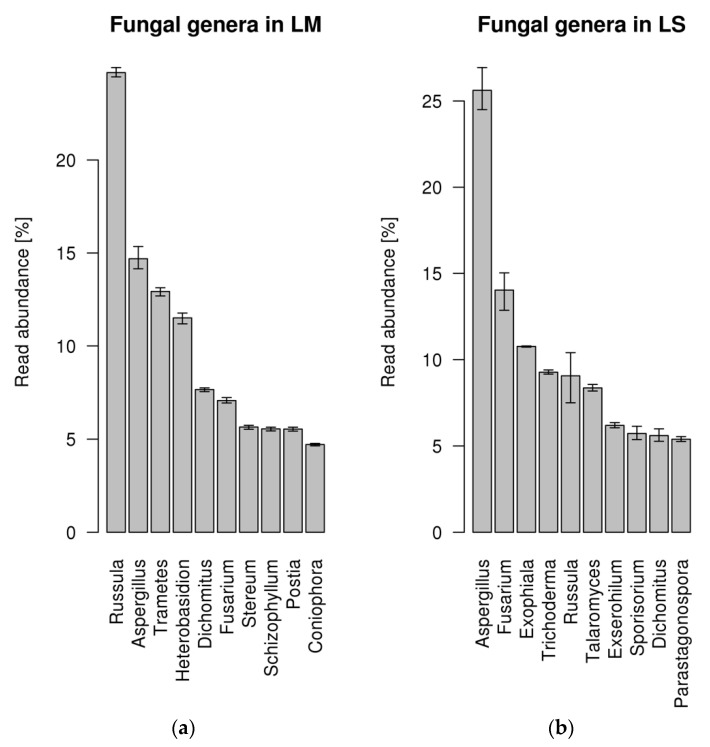
Percentage of fungal reads assigned to different genera in the roots of *Limodorum abortivum*. (**a**) Percentage of fungal reads in mycorrhizal (LM) root samples. (**b**) Percentage of fungal reads in non-mycorrhizal (LS) root samples. The error bar represents the 95% confidence interval of abundance estimate based on three replicates per condition.

Roots of *L. abortivum* were collected in nature where, in addition to mycorrhizal fungi, they interacted with a complex community of bacteria and fungi, as suggested by the range of fungal taxa identified in the metatranscriptome (Figure 1). The 10 most represented fungal genera covered only about 30% of the fungal reads and included both Basidiomycota (i.e., *Russula*, *Trametes*, *Heterobasidion*, *Postia*) and Ascomycota (i.e., *Aspergillus*, *Fusarium*, *Exophiala*). *Russula* was the most abundant fungal genus identified in mycorrhizal roots, confirming that this ECM fungus is the dominant symbiont of *L. abortivum* [32], whereas *Aspergillus*, together with other genera in the Ascomycota, was the most abundant genus in non-mycorrhizal roots (Figure 1). Besides *Russula*, other Basidiomycota mainly in the Polyporales were identified in mycorrhizal but not in non-mycorrhizal *L. abortivum* roots (i.e., *Trametes*, *Heterobasidion*, *Stereum*, *Schizophyllum,* and *Postia*), although their relationship with the plant is unknown. 

**Figure 2:**
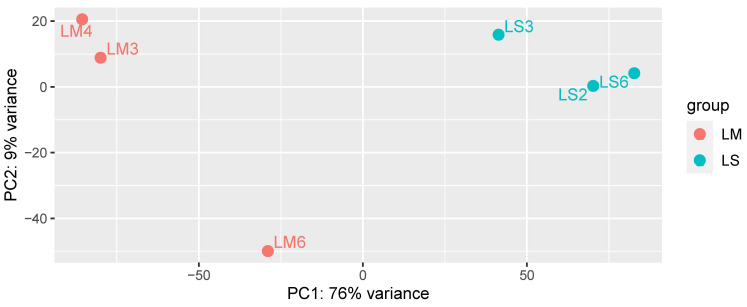
PCA analysis of the complete plant transcriptome in the three biological replicates of mycorrhizal (LM) and non-mycorrhizal (LS) root samples of *Limodorum abortivum*.

When the plant transcriptomic profile was analyzed by PCA, mycorrhizal (LM) and non-mycorrhizal (LS) *L. abortivum* root samples were well separated along the first axis, representing 76% of variance (Figure 2). These results indicate a different plant gene expression profile in root tissues colonized by the mycorrhizal fungal symbiont(s), despite the fact that root samples in nature may display more variability than samples obtained in vitro because they are likely exposed to different environmental conditions and/or microbiota (e.g., see the more distant LM6 sample in Figure 2). Both LM and LS root samples were exposed to a rich microbiota that included non-mycorrhizal fungi, further suggesting that the separation in the PCA (Figure 2) mirrored mycorrhiza-specific plant responses in LM roots.

### 2.3. GO, KEGG Pathway, and CAZyme Enrichment Analyses of Plant Transcripts in Mycorrhizal L. abortivum Roots

**Figure 3:**
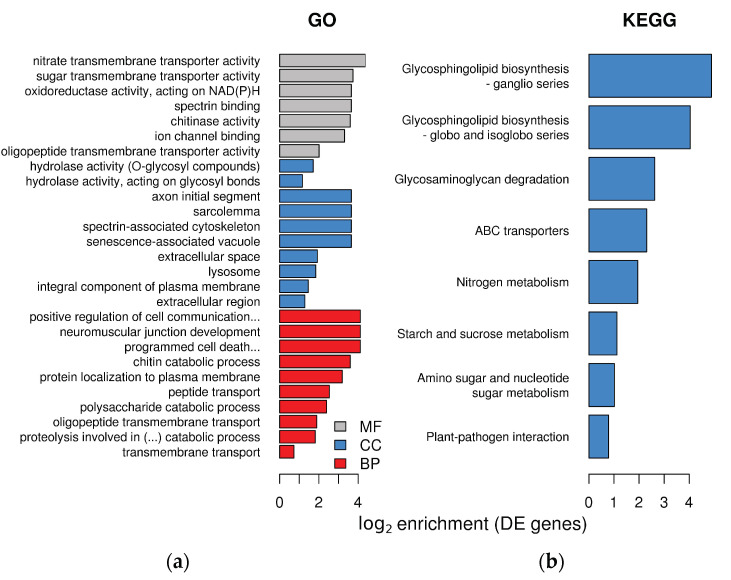
Enrichment in GO terms and KEGG pathways in the plant transcriptome of *Limodorum abortivum* mycorrhizal roots: (**a**) log_2_ enrichment of the 10 most significant terms for each GO category. MF = molecular function, CC = cellular component, BP = biological process; (**b**) enrichment of KEGG pathways in the plant differentially expressed (DE) genes.

Differentially expressed plant transcripts showed significant enrichment for several Gene Ontology (GO) terms in the three GO domains (Figure 3a). In the molecular function domain of GO, *L. abortivum* transcripts showed enrichment in several terms related to hydrolases and peptidases. In the cellular component, we noticed an enrichment for several terms related to synaptic membrane, main axons and similar, which of course are not found in plants. However, these components include integral membrane proteins involved in secretion and vesicle transport in many eukaryotes. For example, two t-SNARE proteins belonging to the syntaxin SYP132 family (TRINITY_DN26107_c1_g3_i10 and TRINITY_DN26107_c1_g3_i4) were significantly upregulated in LM roots (log_2_ FC = 4.6 and 3.6, respectively). In the biological process, several categories related to transport, and specifically transmembrane transport, were overrepresented (Figure 3a). 

Molecular changes in mycorrhizal *L. abortivum* roots, as compared to non-mycorrhizal roots, were further investigated by examining the enrichment of Kyoto Encyclopedia of Genes and Genomes (KEGG) pathways in the plant differentially expressed genes (DEGs). Seven KEGG pathways, mostly related to glycosphingolipids synthesis, nitrogen metabolism, transport, and sugar metabolism were overrepresented (Figure 3b). Differential expression of specific genes related to these metabolic pathways is described in the next paragraphs. 

Interestingly, most of the enriched pathways included upregulated genes (Figure S2), whereas only three enriched pathways included downregulated genes, namely photosynthesis, oxidative phosphorylation, and phenylpropanoid biosynthesis (Figure S3). Genes not differentially expressed in *L. abortivum* roots showed relatively low levels of enrichment in GO categories, with only three enriched GO terms, all in the Biological Process class and with a log_2_ enrichment lower than 1 (Figure S4).

**Figure 4:**
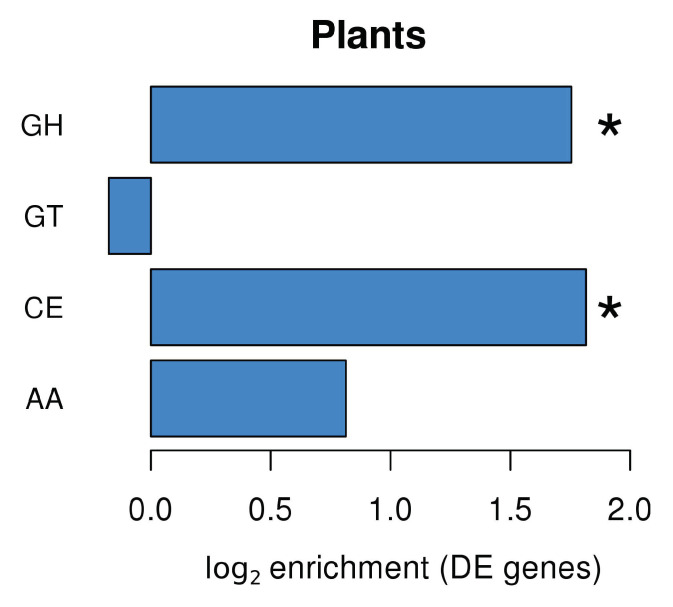
CAZyme families enrichment in *Limodorum abortivum* genes differentially expressed in mycorrhizal roots. GH: glycoside hydrolases; GT: glycosyl transferases; CE: carbohydrate esterases; AA: auxiliary activities. Significant values according to Fisher’s test (*p* < 0.05) are indicated with an asterisk (*).

Plants produce a wide range of enzymes involved in carbohydrate metabolism. Many of these Carbohydrate-Active Enzymes (CAZymes; http://www.cazy.org/) are regulated during plant development and plant responses to biotic and abiotic stresses [34]. Analysis of plant DEGs in *L. abortivum* mycorrhizal roots (LM; Figure 4) led to the identification of several annotated CAZyme domains, with an enrichment of domains corresponding to glycoside hydrolases (GHs) and carbohydrate esterases (CEs). The enrichment analysis showed that GHs were enriched in the upregulated genes (Figure S5), and CEs were enriched in the downregulated genes (Figure S6). In particular, most CEs corresponded to enzymes acting on pectin and were strongly downregulated in LM root samples, like a pectin acetylesterase (TRINITY_DN27496_c1_g1_i8; log_2_ FC = −5.35) and two pectin methylesterases (TRINITY_DN37597_c0_g1_i1; log_2_ FC = −3.3 and TRINITY_DN27102_c2_g1_i1; log_2_ FC = −2.0). Two pectin methylesterase inhibitors (TRINITY_DN27850_c3_g1_i1; log_2_ FC = 2.3 and TRINITY_DN27102_c2_g1_i2; log_2_ FC = 4.4) were by contrast upregulated in LM samples. Among the GHs, the most upregulated glycoside hydrolases were involved in the fungal cell wall modification/degradation and included acidic endochitinases, as well as a putative glucan endo-1,3-beta-glucosidases (Table S2). Chitinases hydrolyze β-1,4-glycosidic bonds and are mostly regarded as part of the plant defense mechanisms induced as a response to colonization by phytopathogenic fungi. However, plant genes mainly encoding class III chitinases are expressed in arbuscule-containing cells in AM, suggesting a potential role in fungal cell wall modification during arbuscule development, as well as in the hydrolysis of chitin-derived elicitors [35,36]. A gene coding for a putative class III endochitinase was among the most upregulated genes in *L. abortivum* mycorrhizal roots (Table 1), in agreement with previous observations in *S. vomeracea* protocorms colonized by *T. calospora* [15]. 

### 2.4. Most Upregulated Transcripts in Mycorrhizal Roots of L. abortivum 

The 25 most upregulated plant DEGs (adjusted *p* < 0.05) are listed in Table 1 and their sequences were further analyzed. Their log_2_ fold-change (FC), when compared with expression in non-mycorrhizal (LS) roots, ranged from 8.26 to 12.02. In detail, the most upregulated gene coded for cucumisin (TRINITY_DN22095_c0_g2_i2), a subtilisin-like endoprotease [37]. Other proteinases, such as cysteine proteases, aspartic proteinase, and a serine carboxypeptidase, were also among the most upregulated *L. abortivum* DEGs (Table 1). In particular, the secreted aspartic proteinase Constitutive Disease Resistance (CDR1), the best Blast match for the *L. abortivum* transcripts (Table 1), plays an important role during the immune response to bacterial pathogens in *Arabidopsis* [38], whereas cysteine proteases are involved in several functions, including senescence and programmed cell death [39]. In addition to these most upregulated genes, several other strongly upregulated proteinase coding genes were identified in mycorrhizal *L. abortivum* roots (Table 1 and Table S2). 

The list of the most upregulated plant genes in mycorrhizal *L. abortivum* roots included genes belonging to different functional categories previously identified in AM roots and in mycorrhizal protocorms of the terrestrial orchid *S. vomeracea* colonized by *T. calospora* [15,21] as well as genes reported to be upregulated in mycorrhizal roots of *O. maculata* colonized by *P. candolleana* [28]. Among these upregulated genes were genes encoding some membrane transporters, such as two bidirectional SWEET sugar transporters (TRINITY_DN27909_c0_g1_i5 and TRINITY_DN26416_c3_g4_i1), a peptide (TRINITY_DN28361_c1_g4_i3) and a copper transporter (TRINITY_DN63579_c0_g1_i1), a gene coding for an acidic endochitinase (TRINITY_DN28711_c0_g3_i3) and a gene coding for a thaumatin-like protein (TRINITY_DN26462_c1_g2_i2). The presence of a gene encoding an early nodulin with a plastocyanin-like domain (TRINITY_DN25136_c5_g6_i3) agrees with previous observations in *S. vomeracea* [15], where a gene coding for a nodulin containing a plastocyanin-like domain (SvNOD1) was expressed only in coil-containing cells [40]. In addition to the top 25 upregulated plant genes in mycorrhizal *L. abortivum* roots, other genes are worth mentioning as they contribute to a better understanding of the molecular and cellular interactions in OM.

Notably, many genes involved in nitrogen metabolism were significantly regulated in mycorrhizal roots of *L. abortivum*. In particular, genes coding for a putative amino acid permease (TRINITY_DN24555_c1_g1_i4; log_2_ FC = 5.8) and a lysine histidine transporter 1- (LHT1; TRINITY_DN28439_c1_g5_i2; log_2_ FC = 4.2) were found to be upregulated, suggesting that amino acids may be a source of nitrogen for adult *L. abortivum* orchids. Two additional amino acid transporter genes were found to be upregulated in mycorrhizal roots of *L. abortivum* (Table S2), but they are unlikely to be involved in symbiotic nitrogen exchange. The first coded for an amino acid permease BAT1 (TRINITY_DN25856_c0_g2_i2; log_2_ FC = 2.9), a membrane transporter likely mediating the transport of gamma-aminobutyric acid from the cytosol to mitochondria [41], whereas the second coded for a cationic amino acid transporter (TRINITY_DN19554_c0_g2_i3; log_2_ FC = 5.4), a permease likely located on the plastid membrane and only expressed in roots in *Arabidopsis* [42]. Genes coding for proteins homologous to members of the NRT1/PTR family were upregulated in mycorrhizal roots of *L. abortivum* (Table 1 and Table S2), and one of them (TRINITY_DN26254_c6_g1_i1; log_2_ FC = 10.19) was among the 25 most upregulated genes. 

Some genes identified in *L. abortivum* roots further support the hypothesis of a supply of organic nitrogen to the host root by the mycorrhizal fungus. In particular, the expression of a high affinity plant nitrate transporter (TRINITY_DN22863_c0_g1_i2) was significantly downregulated (log_2_ FC = −2.5) in *L. abortivum* mycorrhizal roots (Table S2). Different glutamine synthetase (GS) isoforms were upregulated in mycorrhizal *L. abortivum* roots (Table S2), and one of them (TRINITY_DN28208_c3_g1_i5; log_2_ FC = 8.4) was among the 25 most upregulated genes. Cytosolic GS is particularly important for nitrogen remobilization and recycling [43] and strong upregulation of a GS coding gene was already reported in *S. vomeracea* mycorrhizal protocorms [21]. 

Early experiments by Smith [44] indicate that a transfer of carbon occurs in the form of sugars, at least in orchid protocorms. In plants, several sugar transporters, including members of the SWEET family and monosaccharide transporters (MST), are responsible of sugar transport. In *L. abortivum*, two monosaccharide transporters similar to MST3 (TRINITY_DN27753_c2_g4_i1 and TRINITY_DN28768_c4_g4_i7; both log_2_ FC = 3.8) were upregulated in LM samples, together with several transporters of the SWEET family (Table 1 and Table S2). 

## 3. Discussion

Although the last few years have seen an increase in the use of omics approaches to investigate the molecular basis of orchid mycorrhizal symbioses [23,45], information is still patchy mainly due to the complexity of this symbiosis, that involves different developmental stages as well as orchid and fungal symbionts with a range of lifestyles. The absence of *Limodorum abortivum* genome sequence did not make feasible the alignment of transcript short reads directly to a reference genome, thus putatively leading to an incomplete identification of genes. However, through a *de novo* assembly-based method, we have contributed to increase knowledge on the orchid response in a symbiotic system so far unexplored (i.e., a terrestrial mixotrophic adult orchid colonized by an ECM fungus in nature). In addition, comparison of our results with published data indicates that many plant responses identified in *L. abortivum* are conserved not only in other orchid species and developmental stages [16,17,28], but also in AM symbioses [2,3,4], and may mirror a common core of plant genes involved in endomycorrhizal symbioses. 

### 3.1. Disentangling Mycorrhiza Specific and General Responses to Microbes in L. abortivum Roots

Plant-associated microbes can influence gene expression in the host plant by inducing signals that trigger plant immune responses [46] and induce a primed status that helps plants to respond to stress factors [47]. The use of nonsterile non-mycorrhizal roots (LS samples) as reference in our study likely allowed an easier identification of mycorrhiza-specific plant responses with respect to the more general plant responses to microbes. Genes whose expression was not significantly modified in LS and LM root samples were involved in some of these general plant responses (Table S2). Among them, we observed several genes involved in the jasmonic acid (JA) signaling pathway, which is involved in the mediation of plant responses to microorganisms, including the beneficial root-associated microbiota, and may lead to induced systemic resistance [48]. Among genes expressed in both LS and LM roots were also several antioxidative enzymes, pathogenesis related proteins, including chitinases mainly belonging to class V and basic chitinases, and LysM domain receptor kinases likely involved in the recognition of chitin elicitors. The only LysM domain receptor kinase gene that was differentially expressed in LM roots (TRINITY_DN28270_c1_g7_i1; log_2_ FC = −1.88) was actually downregulated. Another interesting example was the expression of phosphate transporters. Transfer of labeled P to mycorrhizal orchid tissues was demonstrated by Cameron et al. [25] and three transcripts coding for putative inorganic phosphate transporters of the PTH1;4 family were identified both in LS and LM samples, but none of them were differentially upregulated in mycorrhizal *L. abortivum* roots. Intriguingly, Zhao et al. [16] observed that a phosphate transporter was upregulated in *C. hybridum* roots inoculated either with the mycorrhizal fungus *Epulorhiza repens* (Tulasnellaceae) or with the non-mycorrhizal root endophyte *Umbelopsis nana*, as compared with uninoculated roots. Thus, it is possible that beneficial components of the *L. abortivum* microbiota may influence expression of genes involved in nutrient acquisition. Such genes would not be identified as DE genes in our transcriptomic analysis. Overall, these observations suggest that the comparison of gene expression between LM and LS samples under natural conditions may have favored the identification of mycorrhiza-specific plant responses rather that general plant responses to the associated microbiota.

### 3.2. Genes Potentially Involved in Plant Cell Wall Remodeling and Interface Construction during Fungal Accommodation

Pectins are an important component of the plant cell wall and pectin remodeling is a central issue to understand the fundamental role of homogalacturonan-type pectins during plant growth and plant responses to environmental changes [49]. Pectin is synthesized in a highly methyl esterified form and is de-esterified within the wall by pectin methyl esterases (PMEs), thus exposing carboxyl residues that can be cross-linked by calcium [50]. The activity of PMEs is modulated by a family of proteinaceous inhibitors known as PME inhibitors (PMEIs), and their interplay can thus strengthen or loosen the cell wall [51]. Among the CAZymes differentially expressed in *L. abortivum*, some PMEs were strongly downregulated and PMEIs were upregulated in mycorrhizal roots, indicating that pectin in the peripheral cell wall and/or symbiotic interface is mostly in a highly methylated form in mycorrhizal roots. As a high degree of methyl esterification (DM) leads to a softer and more pliant gel in vitro and *in planta*, permissive of cell growth, we may speculate that pectin softening may be related with intracellular accommodation of fungal coils in the mycorrhizal roots and/or with cell expansion. Cell wall loosening is also supported by the upregulated expression of two expansin coding genes in mycorrhizal roots (TRINITY_DN3991_c0_g1_i1; log_2_ FC = 5.8 and TRINITY_DN23405_c0_g1_i3; log_2_ FC = 4.9). Pectin remodeling also plays an important role in biotic interactions, and plants with a higher DM in the cell walls due to PMEI overexpression showed in general increased resistance to fungal and bacterial pathogens [51]. Considering the role of oligogalacturonides as signaling molecules and the fact that oligogalacturonides with different degrees of esterification are expected to be released depending on the circumstances [52], our findings raise the question of whether pectin fragments might be involved or not in eliciting plant responses during OM symbiosis, as suggested in ECM [53]. 

Transcripts coding for subtilisin-like serine proteases were highly upregulated in the transcriptome of *L. abortivum* mycorrhizal roots. Most subtilisin-like serine proteases, or subtilases (SBTs), are targeted to the plant cell wall, where they can contribute to regulate cell wall organization and structure [54]. Intriguingly, a loss-of-function mutant for a SBT essential for normal seed mucilage release had increased PME activity in the seeds, suggesting that this SBT acts as a repressor of PME activity [55]. Subtilases can also function in mutualistic and pathogenic plant–microbe interactions, as they can be modulators of the plant immune responses. Several SBT genes are induced during formation of AM and nodule symbioses in the legume *Lotus japonicus* [56,57], and their expression has been found to be dependent on the common symbiotic pathway [58], a signaling pathway conserved in all land plants forming endosymbiosis [59]. 

Two strongly upregulated genes in mycorrhizal *L. abortivum* roots coded for syntaxin-132 (SYP132) proteins, also reported in mycorrhizal root of other orchid species [16,28]. Syntaxins have been extensively investigated in the AM symbiosis [60] and the SYP132α isoform, in particular, was demonstrated by knockdown mutations to be essential for arbuscule formation in *Medicago truncatula* [60]. Given the localization of SYP132 on the perisymbiotic plant membrane surrounding functional arbuscule branches, this t-SNARE protein, with a putative role in vesicle trafficking, has been suggested to be instrumental for the formation of a functioning plant–fungus interface. Although the localization of the SYP132 proteins in OM roots is currently unknown, it is tempting to speculate that a common exocytotic pathway is shared by AM and OM symbioses. Interestingly, another upregulated gene in mycorrhizal roots of *L. abortivum* coded for a putative synaptotagmin, which is involved in membrane fusion and can bind to t-SNAREs proteins as well as to v-SNAREs. Synaptotagmin was identified as an important component in the formation of the symbiotic membrane interface in root nodules [61], and an *O. maculata* homolog gene was reported to be upregulated in colonized adult roots [28]. 

### 3.3. Nutrient Exchanges in the Mycorrhizal Roots of an Adult Orchid

Recent findings in mycoheterotrophic *S. vomeracea* protocorms [17] suggest that the mycorrhizal fungus *T. calospora* provides the host with amino acids, likely as a source of both nitrogen and carbon. Genes coding for a putative amino acid permease and a lysine histidine transporter 1 (LHT1) were also upregulated in *L. abortivum* mycorrhizal roots. Despite the name, LHT1 can transport a broad range of amino acids, with preference for neutral and acidic amino acids, as observed in rice [62]. Upregulated genes coding for amino acid transporters and for a LHT1 have been also reported in mycorrhizal roots of the epiphytic orchid *C. hybridum* [16] and of the terrestrial orchid *O. maculata* [28]. Overall, these observations suggest that orchids receive from their mycorrhizal fungal partners organic nitrogen in the form of amino acids irrespective of the plant developmental stage, trophic strategy of the adult plant, and type of fungal partner. The expression pattern of the *S. vomeracea LHT1* in mycorrhizal protocorm cells indicates a localized expression in cells containing viable fungal hyphae, suggesting a role in amino acid transfer across an intact plant–fungus membrane interface [21]. The LjLHT1.2 gene, encoding a LHT1-type amino acid transporter, was also consistently expressed in cortical cells of AM roots, where transcripts were localized mainly in arbusculated cells but also in the noncolonized cells of the root cortex [63].

Different NRT1/PTR family members were upregulated in *L. abortivum* roots. Members of this protein family display sequence and structural homologies with peptide transporters involved in the uptake of di- or tripeptides [63,64] and may be involved in N transfer from the fungus to the host plant. Members of the NRT1/PTR family were also upregulated in mycorrhizal roots of *C. hybridyum* [16], *O. maculata* [28], and in *S. vomeracea* protocorms [21], as well as in the AM symbiosis [3], where a PTR coding gene was expressed in arbusculated cells but not in noncolonized cells of mycorrhizal roots. However, members of this transporter family have been found to translocate several other substrates, in addition to peptides, such as nitrite, chloride, glucosinolates, auxin, abscisic acid, jasmonates, and gibberellins [65]. Their role in the mycorrhizal symbiosis remains therefore uncertain.

If amino acids seem to be the main source of nitrogen for mycorrhizal orchids, their carbon backbone may also represent an important source of carbon for the host plant, especially during the juvenile developmental stages and for mycoheterotrophic and mixotrophic species. Upregulated transcripts coding for SWEET transporters were reported in mycorrhizal roots of the putative mixotrophic *O. maculata* [28], in mycorrhizal mycoheterotrophic *S. vomeracea* protocorms [21], and in the mycorrhizal roots of *E. helleborine* albino variants [30]. It is worth noting that SWEET transporters were not upregulated in the mycorrhizal roots of the photosynthetic orchid *C. hybridum* [16]. These data indicate that SWEET transporters may play important roles in mycoheterotrophic developmental stages as well as in adult plants of orchid species that rely on the mycorrhizal fungal symbiont for carbon (i.e., mycoheterotrophic and mixotrophic orchids). The role and localization of these different sugar transporters require further investigations, but it is interesting that several MST and SWEET transporters were also regulated during the parasitic interaction of plant species that directly obtain carbon from their host plants [66].

## 4. Materials and Methods

### 4.1. Sampling and Preparation of Biological Materials

Transcriptional differences between mycorrhizal and non-mycorrhizal *Limodorum abortivum* roots were evaluated in samples collected in a single site in the Piedmont Region in North-West Italy (Monte San Giorgio, Torino, Italy). Plants growing on this site were shown to harbor a dominant mycorrhizal symbiont phylogenetically close to the ECM basidiomycete *Russula delica* (site IT1B in [32]). Collected root samples were thoroughly cleaned from soil debris and rinsed under tap water. Mycorrhizal roots (LM) are easily recognizable because they are thicker and darker in color than non-mycorrhizal roots (LS). Fresh hand-made sections were prepared to verify the mycorrhizal status at increasing distances from the root apex, and root segments (about 1 cm) were flash frozen in liquid nitrogen and stored at −80 °C until use. 

### 4.2. RNA Extraction, Library Preparation, and Sequencing

Frozen root segments from different individuals were ground in liquid nitrogen using sterile mortar and pestles. Total RNA was extracted from samples following the protocol described by Chang et al. [67]. Genomic DNA was removed using the Turbo DNA-freeTM reagent (Ambion, Austin, TX, USA), according to the manufacturer’s instructions. Three biological replicates for each condition (mycorrhizal/non-mycorrhizal) were used. The quality of the isolated RNA was determined using a Nanodrop 1000 spectrophotometer (Thermo Scientific, Waltham, MA, USA) and a Bioanalyzer 2100 (Agilent, Palo Alto, CA, USA). Samples further processed had a RIN score ranging from 6.8 to 8.7 for mycorrhizal samples and from 7.8 to 8.0 for non-mycorrhizal samples, an OD 260 to 280 nm ratio between 1.8 and 2.1, and a 28S/18S ratio within 1.5–2.

cDNA libraries were constructed using 2 µg of total RNA and following the TruSeq RNA Sample preparation v.2 kit instructions (Low throughput protocol, Illumina, Inc., San Diego, CA, USA). Briefly, total RNA samples were polyA-enriched, reverse-transcribed, and double-stranded cDNA synthesized. TruSeq adapters were attached to double-stranded cDNA. Enrichment in fragments containing TruSeq adapters on both ends were performed using PCR. The quantity and quality of the enriched libraries were validated using Nanodrop 1000 and Bioanalyzer 2100, respectively. The libraries were normalized to 10 nM of amplicons in 10 mM Tris-HCl, pH 8.5 containing 0.1% Tween 20. Barcoded amplicons (library) were spread over one Illumina HiScan 1000 lane. The TruSeq PE Cluster Kit v3-cBot-HS (Illumina, Inc., San Diego, CA, USA) was used for cluster generation using 2 pM of pooled normalized libraries on the cBOT. Sequencing was performed on the Illumina HiScan 1000 to yield paired-end reads of 2 × 101 bases using the TruSeq SBS Kit v3-HS (Illumina, Inc., San Diego, CA, USA). Reads were quality-checked with Fastqc (http://www.bioinformatics.babraham.ac.uk/projects/fastqc/), which computes various quality metrics for the raw reads. 

### 4.3. Bioinformatics Analysis

Adapter removal and trimming of bases with phred quality score lower than 10 were performed using bbduk (http://sourceforge.net/projects/bbmap/). Transcriptome assembly was performed using the reads from all replicates and all conditions using Trinity [68] with default parameters. Reads from each sample were then aligned against the assembled transcriptome using Hisat2 [69], generating a sample-specific transcriptome. The assembler Stringtie was used to refine the individual transcriptome based on realignment of the reads on the transcriptome, and to merge and harmonize each sample into a unique consensus transcriptome [70], on which subsequent alignment-based analysis were performed. Taxonomic classification of the reads and of the reconstructed transcripts was performed using kraken2 [71] against the nt database. Taxonkit [72] was used to specifically select and group the reads and the transcripts belonging to plants (Streptophyta) and fungi (Ascomycota or Basidiomycota). Htseq-count [73] was used to count reads mapping on the genes classified as belonging to plants or fungi. Differential expression analysis was performed separately in plant and fungal transcripts using DEseq2 [33,74]. Taxonkit [72] was used for specifically selecting and grouping the contigs belonging to plants (Streptophyta) and fungi (Ascomycota or Basidiomycota). All the downstream analysis was performed for the two groups of organisms separately. A principal component analysis (PCA) was performed on normalized read counts, to assess differences among sample replicates and between conditions. 

Functional annotation of the reconstructed transcriptome was performed relying on Gene Ontology [75] and KEGG [76]. In detail, blastx [77] against UniProt database was used to classify the reconstructed transcripts; GO annotation of the UniProt database was retrieved from http://current.geneontology.org/annotations/index.html, so that each transcript mapping to a UniProt term also inherited its GO annotation. In addition, transcripts were mapped to the corresponding KEGG term using the R package KEGGREST [78]. KEGG terms were summarized to KEGG pathways using the R package KEGGREST [78]. Classification of transcripts as Carbohydrate Active Enzymes (CAZymes) was performed using dbCAN2 [79]; dbCAN2 can use three different classification tools. As recommended by these authors, only transcripts identified as CAZymes by at least two tools were considered as CAZymes. Enrichment for GO, KEGG, and CAZyme terms was assayed using Fisher’s test. Enrichment analysis was performed to test enrichment in all differentially expressed genes. Additional analyses were performed to test enrichment in upregulated and downregulated genes separately, and on genes showing no differential expression. 

Among the differentially expressed plant genes (adjusted *p* < 0.05), the 25 showing the highest log_2_ fold-change were further characterized through Blastx against the Refseq database and conserved and functional motifs were considered.

## 5. Conclusions

We investigated the transcriptomic profile of mycorrhizal roots of the mixotrophic orchid *L. abortivum* grown in nature. Comparison with available gene expression data in three other orchid species (*S. vomeracea*, *O. maculata*, and *C. hybridum*) indicate that many molecular markers are shared in mycorrhizal orchid tissues despite the different plant trophic strategy (photosynthetic, mycoheterotrophic or mixotrophic), developmental stage (protocorms or adult plants), habitat (tropical or temperate, terrestrial or epiphytic), and type of fungal partner (rhizoctonia-like, ECM or wood decomposing fungi). Many molecular markers (such as components involved in vesicle trafficking, amino acid transporters, and subtilases) were also reported in AM roots (and even legume nodule) symbioses. Thus, they may represent components of a common symbiotic toolkit shared by different endosymbioses. The analysis of a wider range of orchid species with diverse trophic strategies may help in understanding whether the OM specificities depend on fine-tuned regulation of common cell components, or whether specific additional genes are involved. 

## Figures and Tables

**Table 1:** List of the 25 most upregulated plant DEGs identified in this study.

Sequence ID	log_2_ Fold Change	Putative Function (Blastx, Refseq)	Plant Species	Query Cov. (%)	Ident. (%)	E-Value
TRINITY_DN22095_c0_g2_i2	12.02	Cucumisin-like	*Dendrobium catenatum*	85	69.68	0.0
TRINITY_DN27918_c0_g1_i6	11.02	Senescence-specific cysteine protease SAG39-like	*Populus euphratica*	72	79.52	2e-88
TRINITY_DN21619_c0_g1_i2	10.93	4,5-DOPA dioxygenase extradiol	*Dendrobium catenatum*	53	86.98	1e-98
TRINITY_DN24828_c2_g2_i3	10.54	Putative beta-glucosidase	*Populus alba*	46	82.50	2e-87
TRINITY_DN26254_c6_g1_i1	10.19	Protein NRT1/ PTR FAMILY 8.2-like	*Dendrobium catenatum*	75	65.88	0.0
TRINITY_DN27041_c6_g2_i1	10.00	Uncharacterized protein	*Dendrobium catenatum*	52	52.40	3e-57
TRINITY_DN28711_c0_g3_i3	9.54	Acidic endochitinase-like	*Dendrobium catenatum*	62	80.41	1e-166
TRINITY_DN26462_c1_g2_i2	9.71	Thaumatin-like protein 1b	*Phalaenopsis equestris*	51	83.56	3e-115
TRINITY_DN25136_c5_g6_i3	9.53	Early nodulin-like protein 2 (plastocyanin-like)	*Phalaenopsis equestris*	32	76.70	2e-50
TRINITY_DN25901_c2_g1_i9	9.44	Putative calcium-binding protein CML31	*Dendrobium catenatum*	33	67.13	2e-56
TRINITY_DN22366_c0_g1_i1	9.22	Serine carboxypeptidase II-3	*Dendrobium concatenatum*	71	75.88	0.0
TRINITY_DN25614_c3_g3_i8	9.16	Oryzain gamma chain-like (putative cysteine peptidase)	*Dendrobium catenatum*	43	83.13	0.0
TRINITY_DN38600_c0_g1_i1	9.06	Aspartic proteinase CDR1-like	*Dendrobium catenatum*	88	79.73	0.0
TRINITY_DN28212_c0_g1_i1	9.02	Aspartic proteinase CDR1-like	*Phalaenopsis equestris*	88	80.28	0.0
TRINITY_DN29286_c2_g2_i7	8.99	Alpha-humulene synthase-like	*Phalaenopsis equestris*	82	67.58	2e-82
TRINITY_DN26369_c2_g2_i2	8.61	Uncharacterized protein	*Phalaenopsis equestris*	59	58.79	3e-47
TRINITY_DN63579_c0_g1_i1	8.55	Copper transporter 6-like	*Dendrobium catenatum*	38	72.36	5e-47
TRINITY_DN27909_c0_g1_i5	8.54	Bidirectional sugar transporter SWEET4-like	*Phalaenopsis equestris*	32	95.24	3e-29
TRINITY_DN23758_c0_g1_i1	8.50	Heparanase-like protein 3	*Dendrobium catenatum*	70	68.65	2e-177
TRINITY_DN19642_c0_g1_i1	8.50	Glucan endo-1,3-beta-glucosidase	*Dendrobium catenatum*	78	72.67	7e-167
TRINITY_DN20494_c0_g1_i6	8.49	EG45-like domain containing protein	*Populus trichocarpa*	30	49.49	2e-20
TRINITY_DN28208_c3_g1_i5	8.39	Glutamine synthetase nodule isozyme	*Phoenix dactylifera*	32	87.78	1e-172
TRINITY_DN25633_c3_g1_i10	8.28	Beta-hexosaminidase 3	*Dendrobium catenatum*	63	87.21	0.0
TRINITY_DN26416_c3_g4_i1	8.27	Bidirectional sugar transporter SWEET4-like	*Dendrobium catenatum*	81	86.79	1e-56
TRINITY_DN28361_c1_g4_i3	8.26	Oligopeptide transporter 1-like	*Dendrobium catenatum*	69	76.14	5e-123

## Data Availability

Sequencing reads and read counts per gene are available in GEO (GSE159700). Fasta file of raw transcriptome assembly, gtf of refined transcriptome assembly, and results of DE analysis are available on Open Science Foundation, with DOI 10.17605/OSF.IO/35XUP, and can be accessed at https://osf.io/35xup/. Shell scripts and functions used in this work are freely available at https://github.com/fabiomarroni/limodorum.

## Supplementary Materials

The following are available online at https://www.mdpi.com/2223-7747/10/2/251/s1, Figure S1: Percentage of Streptophyta reads assigned to different genera, Figure S2: Log2 enrichment of GO terms and KEGG pathways in genes upregulated in Limodorum abortivum mycorrhizal roots. Left panel: log2 enrichment of the most significant terms for each GO category. MF = molecular function, CC = cellular component, BP = biological process (max 10 terms per category). Right panel: log2 enrichment of KEGG pathways, Figure S3: Log2 enrichment of GO terms and KEGG pathways in genes downregulated in Limodorum abortivum mycorrhizal roots. Left panel: log2 enrichment of the 10 most significant terms for each GO category. MF = molecular function, CC = cellular component, BP = biological process (max 10 terms per category). Right panel: log2 enrichment of KEGG pathways, Figure S4: Log2 enrichment of GO terms and KEGG pathways in genes showing no variation of expression in Limodorum abortivum mycorrhizal roots compared to the control. Left panel: log2 enrichment of the most significant terms for each GO category. MF = molecular function, CC = cellular component, BP = biological process (max 10 terms per category). Right panel: log2 enrichment of KEGG pathways, Figure S5: CAZyme families enrichment in Limodorum abortivum genes upregulated in mycorrhizal roots. GH: glycoside hydrolases; GT: glycosyl transferases; CE: carbohydrate esterases; AA: auxiliary activities. Significant values according to Fisher’s test (*p* < 0.05) are indicated with an asterisk (*), Figure S6: CAZyme families enrichment in Limodorum abortivum genes downregulated in mycorrhizal roots. GH: glycoside hydrolases; GT: glycosyl transferases; CE: carbohydrate esterases; AA: auxiliary activities. Significant values according to Fisher’s test (*p* < 0.05) are indicated with an asterisk (*), Table S1: Summary statistics of reads production and alignments, Table S2: Complete list of plant genes in the mycorrhizal root transcriptome of *L. abortivum* (excel file).
