# Supplementary material for: A Transcriptomic Approach Provides Insights on the Mycorrhizal Symbiosis of the Mediterranean Orchid Limodorum abortivum in Nature
Source: Plants (Basel). 2021 Jan 28;10(2):251. doi: 10.3390/plants10020251 (PMC7911150; doi:10.3390/plants10020251)
Supplement: Supplementary file 1 [file plants-10-00251-s001.zip › SM/Table_S1.docx]

**Table S1.** Summary statistics of reads production and alignments.

| Replicate ID# | Raw reads | Trimmed reads | Aligned | Uniquely aligned | Uniquely aligned to plant transcripts | Uniquely aligned to fungal transcripts |
| --- | --- | --- | --- | --- | --- | --- |
| LM3 | 13718086 | 13688003 | 11504790 | 6019195 | 4265985 | 750411 |
| LM4 | 13154728 | 13125510 | 11123579 | 5834757 | 3913284 | 1066576 |
| LM6 | 12457097 | 12431983 | 10466789 | 5364549 | 4483370 | 270948 |
| LS2 | 13977088 | 13935605 | 11625568 | 5809054 | 5053063 | 128347 |
| LS3 | 13921017 | 13885425 | 11781533 | 5195580 | 4445546 | 243333 |
| LS6 | 12641329 | 12606692 | 10862322 | 4748078 | 4058366 | 201202 |
