# Supplementary figures and images for: A Transcriptomic Approach Provides Insights on the Mycorrhizal Symbiosis of the Mediterranean Orchid Limodorum abortivum in Nature
Source: Plants (Basel). 2021 Jan 28;10(2):251. doi: 10.3390/plants10020251 (PMC7911150; doi:10.3390/plants10020251)

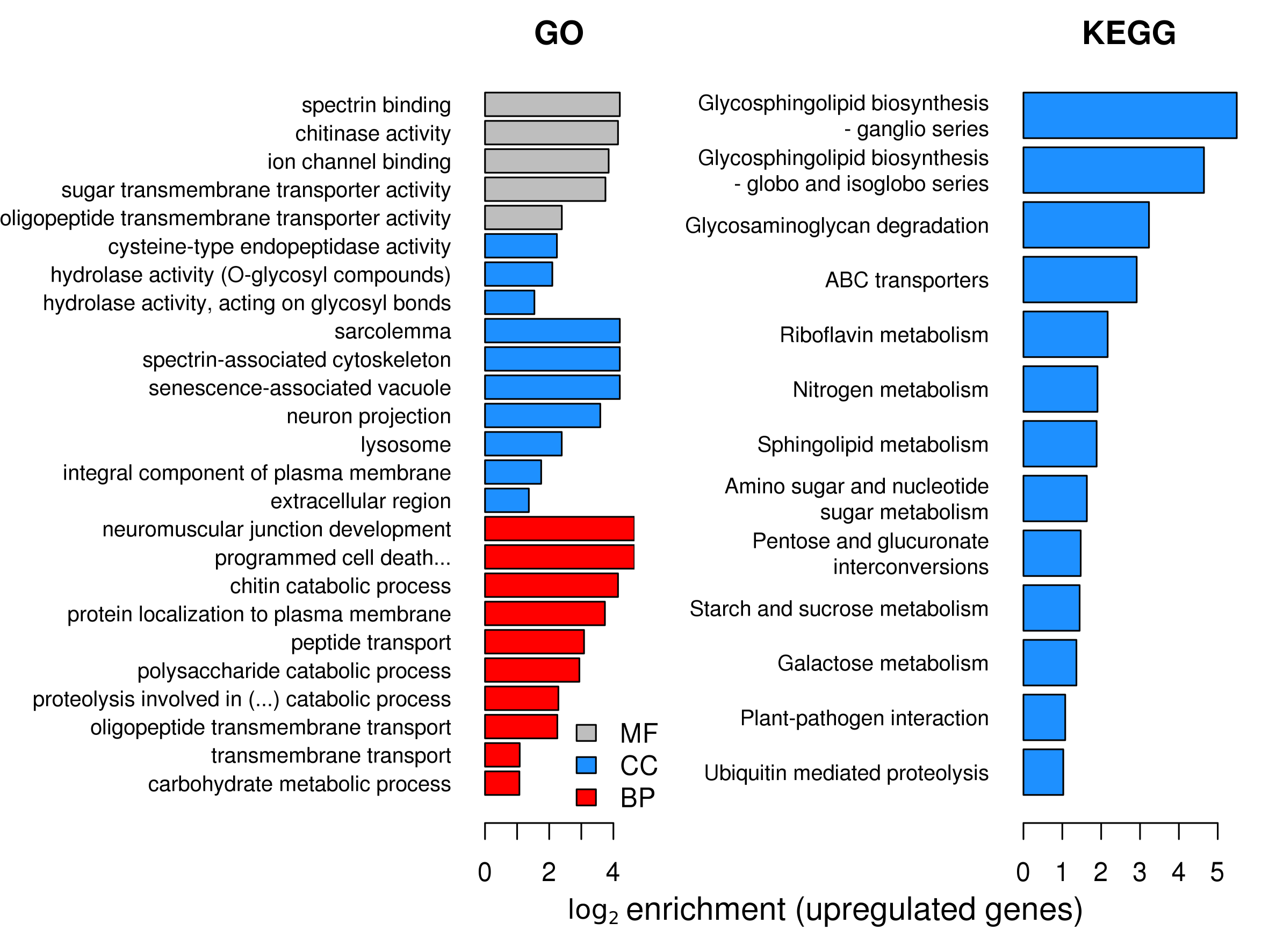

Supplement: Supplementary file 1 [file plants-10-00251-s001.zip › SM/Fig_S2.png.png]

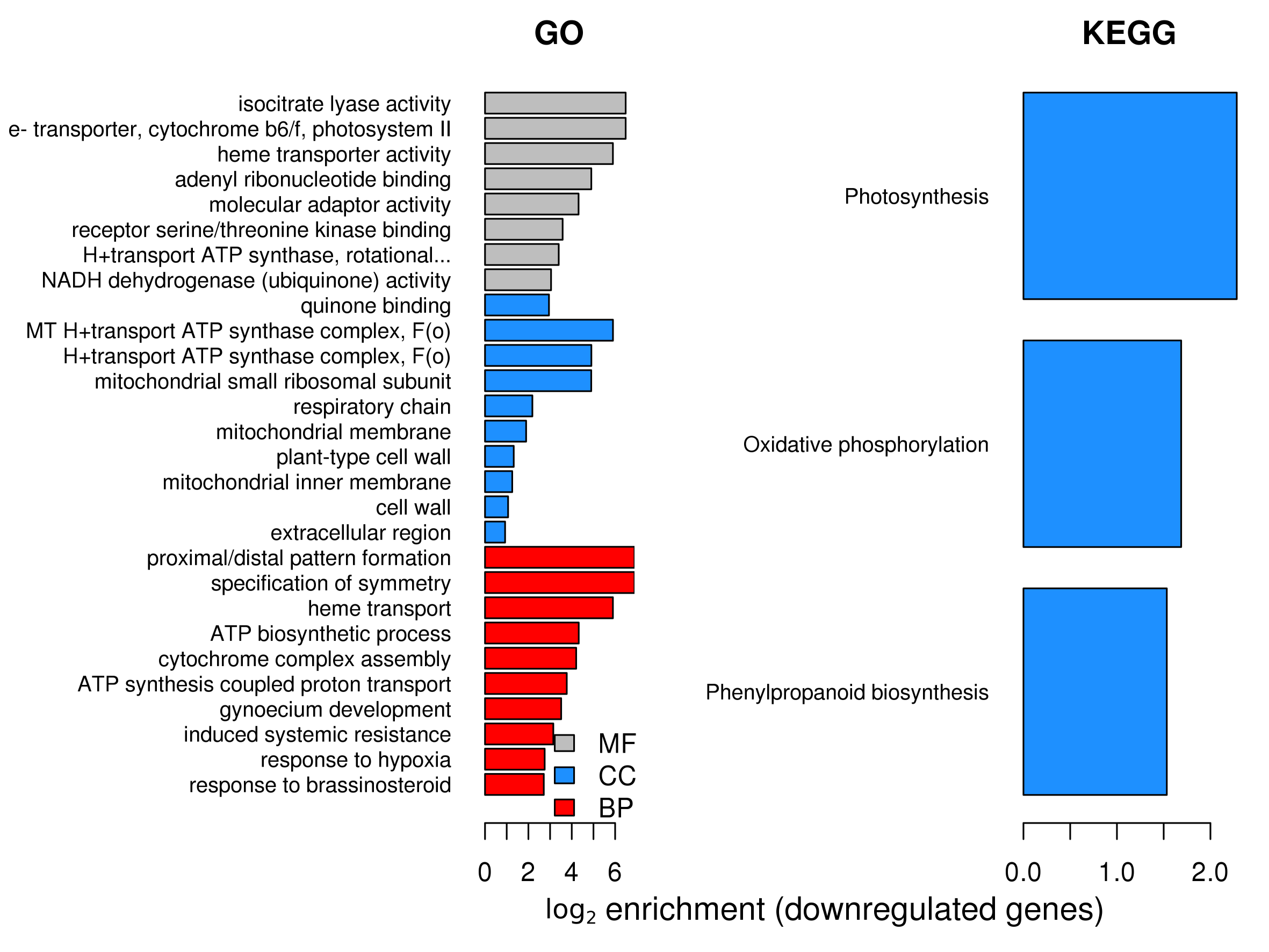

Supplement: Supplementary file 1 [file plants-10-00251-s001.zip › SM/Fig_S3_png.png]

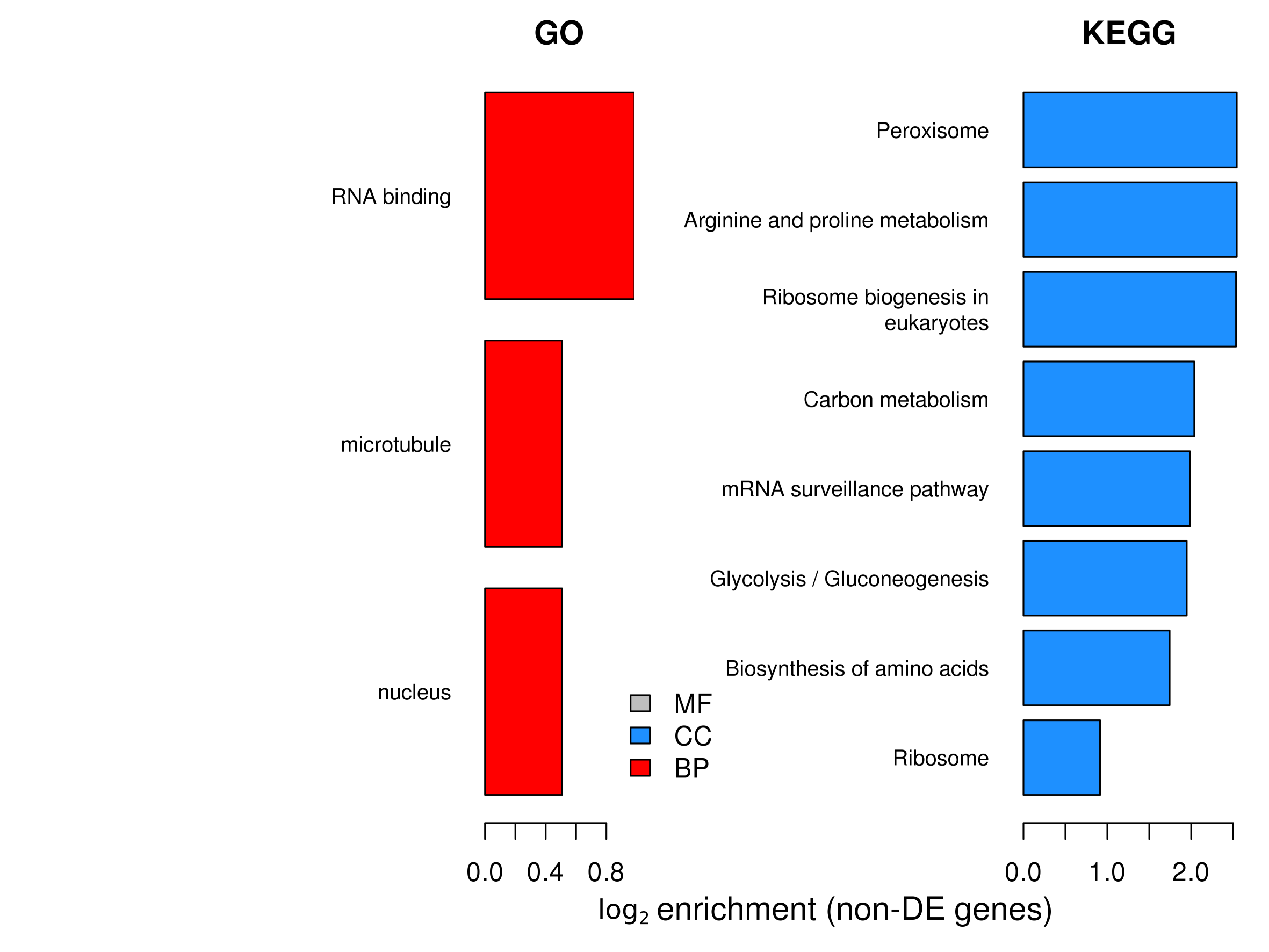

Supplement: Supplementary file 1 [file plants-10-00251-s001.zip › SM/Fig_S4.png.png]

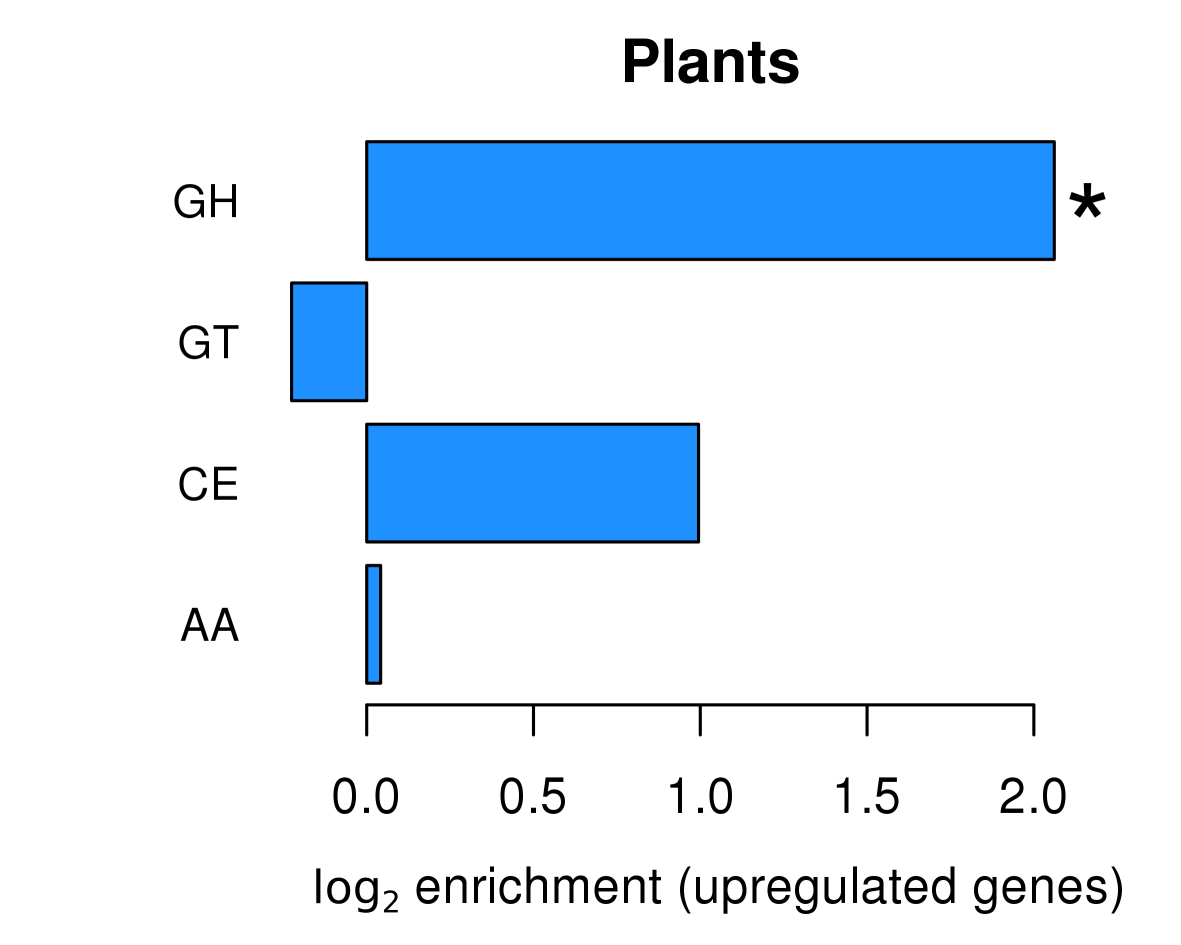

Supplement: Supplementary file 1 [file plants-10-00251-s001.zip › SM/Fig_S5.png.png]

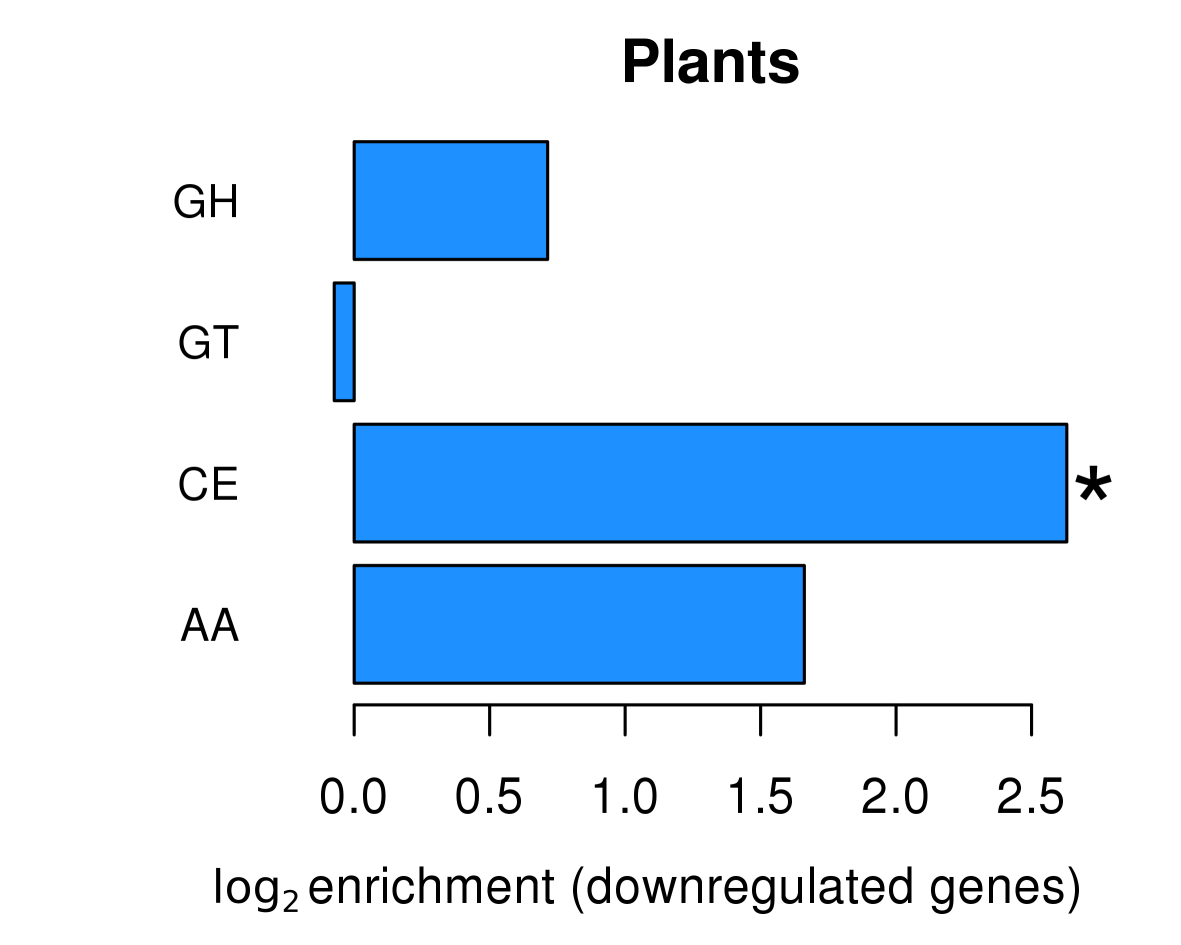

Supplement: Supplementary file 1 [file plants-10-00251-s001.zip › SM/Fig_S6.png.png]

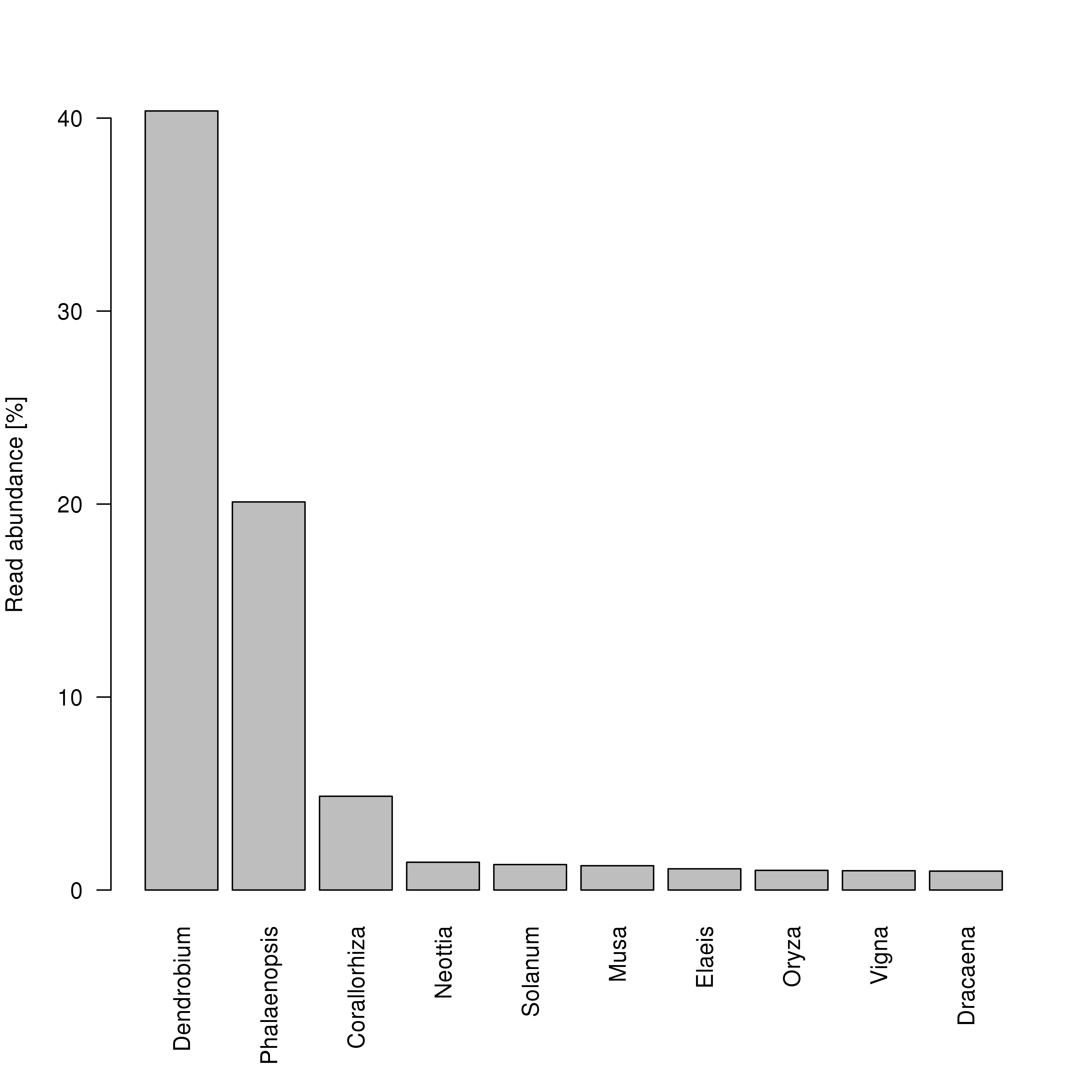

Supplement: Supplementary file 1 [file plants-10-00251-s001.zip › SM/Figure_S1.png]
